# Evaluating the predictive value of biomarkers for efficacy outcomes in response to pertuzumab- and trastuzumab-based therapy: an exploratory analysis of the TRYPHAENA study

**DOI:** 10.1186/bcr3690

**Published:** 2014-07-08

**Authors:** Andreas Schneeweiss, Stephen Chia, Roberto Hegg, Christoph Tausch, Rahul Deb, Jayantha Ratnayake, Virginia McNally, Graham Ross, Astrid Kiermaier, Javier Cortés

**Affiliations:** 1 National Center for Tumor Diseases, University Hospital, Im Neuenheimer Feld 460, 69120 Heidelberg, Germany; 2 British Columbia Cancer Agency - Vancouver Centre, University of British Columbia, 2329 W Mall, Vancouver, BC V6T 1Z4, Canada; 3 Hospital Pérola Byington, Avenida Brigadeiro Luís Antônio, 683, São Paulo, SP 01317-000, Brazil; 4 Breast Center, Seefeldstrasse 214, 8008 Zürich, Switzerland; 5 Department of Cellular Pathology, Derby Hospitals NHS Foundation Trust, Uttoxeter New Road, Derby DE22 3NE, UK; 6 Roche Products Limited, 1 Falcon Way, Welwyn Garden City AL7 1TW, UK; 7 Genentech Early Research and Development, Basel, Switzerland; 8 Vall d’Hebron University Hospital, Vall d’Hebron Institute of Oncology (VHIO), Passeig Vall d'Hebron 119, 08035 Barcelona, Spain

## Abstract

**Introduction:**

Molecular markers that predict responses to particular therapies are invaluable for optimization of patient treatment. The TRYPHAENA study showed that pertuzumab and trastuzumab with chemotherapy was an efficacious and tolerable combination for patients with human epidermal growth factor receptor 2 (HER2)-positive breast cancer in the neoadjuvant setting. We analyzed whether particular biomarkers correlated with the responses observed and therefore may predict outcomes in patients given pertuzumab plus trastuzumab.

**Methods:**

We describe the analysis of a panel of biomarkers including HER2, human epidermal growth factor receptor 3 (HER3), epidermal growth factor receptor (EGFR), phosphatase and tensin homolog (PTEN), and phosphatidylinositol-4,5-bisphosphate 3-kinase catalytic subunit alpha (PIK3CA) by qRT-PCR, immunohistochemistry (IHC), fluorescence *in situ* hybridization (FISH), enzyme-linked immunosorbent assay (ELISA), and PCR-based mutational analyses as appropriate. For each marker analyzed, patients were categorized into ‘low’ (generally below median) or ‘high’ (generally above median) subgroups at baseline and post-treatment.

**Results:**

Correlation of marker subgroups with the achievement of a pathological complete response (pCR) (ypT0/is) was analyzed. HER2 protein and mRNA expression levels were associated with pCR rate in two of the three study arms and the pooled analyses. Correlations of biomarker status with pCR occurred in one individual arm only and the pooled analyses with EGFR and PTEN; however, interpretation of these results is limited by a strong imbalance in patient numbers between the high and low subgroups and inconsistency between arms. We also found no association between expression levels of *TOP2A* and pCR rate in either the anthracycline-containing or free arms of TRYPHAENA.

**Conclusions:**

According to these analyses, and in line with other analyses of pertuzumab and trastuzumab in the neoadjuvant setting, we conclude that HER2 expression remains the only marker suitable for patient selection for this regimen at present.

**Trial registration:**

The TRYPHAENA study was registered with ClinicalTrials.gov, NCT00976989, on September 14 2009.

## Introduction

Human epidermal growth factor receptor 2 (HER2) overexpression or amplification has previously been identified as a poor prognostic factor in breast cancer patients [1,2]. Novel HER2-targeting agents have, however, improved outcomes in patients with HER2-positive breast cancer to a comparable level to those in patients with HER2-negative disease [3], if not greater relative to certain subtypes (for example, basal-like breast cancers).

HER2-targeted therapies are the mainstay of treatment of HER2-overexpressing or gene-amplified metastatic breast cancer [4]. The addition of trastuzumab (Herceptin™, Roche, Switzerland) to a taxane improved overall survival in patients with HER2-overexpressing metastatic breast cancer [5]. The CLEOPATRA study showed that the addition of pertuzumab (PERJETA™, Roche, Switzerland) to trastuzumab plus taxane further increased progression-free survival (PFS) [6] as well as significantly improving overall survival (OS) when compared with trastuzumab plus a taxane alone [7]. Like trastuzumab, pertuzumab is a HER2-targeted humanized monoclonal antibody; however, it binds to a unique epitope in the dimerization domain and has a complementary mode of action to trastuzumab [8,9]. Thus, HER2 is currently the only validated biomarker for treatment with trastuzumab or pertuzumab in metastatic breast cancer (MBC) [10,11].

In addition to HER2, a number of components from the HER signaling pathways and other signaling pathways have previously been suggested to predict responsiveness (or lack of) to HER-targeted agents, including phosphatase and tensin homolog (PTEN), [12-14], Akt [15], p95^HER2^[16], c-Myc [17], and various growth factors and their receptors such as insulin-like growth factor (IGF-1) [18]. These studies concluded that expression levels of these pathway components had an impact on outcomes and often correlated with resistance to trastuzumab. Conflicting clinical data exist on the potential predictive value of serum extracellular domain (ECD) HER2 levels for the outcome of treatment with HER2-targeted agents [19]. Fornier *et al*. [19] showed that (serum/shed) human epidermal growth factor receptor 2 (sHER2) levels correlated with response rate; however, Lennon *et al*. [20] saw no clear relationship between baseline sHER2 levels and tumor response. Additional data are therefore required to clarify the role of sHER2 as a marker of response.

The TRYPHAENA study, a randomized, multicenter, multinational phase II study, showed that pertuzumab in combination with trastuzumab given either concomitantly or sequentially with standard anthracycline-based chemotherapy or concomitantly with a non-anthracycline-based chemotherapy regimen, is well tolerated and efficacious as neoadjuvant therapy for patients with locally advanced, inflammatory, or early stage HER2-positive breast cancer [21]. An exploratory analysis of biomarkers to determine whether biomarker expression correlated with response to trastuzumab- plus pertuzumab-based regimens was carried out. The biomarkers investigated included markers of the expression and activation of HER-family receptors and related receptor tyrosine kinases, ligands, and downstream signaling components [22]. The biomarkers measured were HER2 (protein and mRNA), HER3 (protein and mRNA in tumor tissue), epidermal growth factor receptor (EGFR) (protein and mRNA in tumor tissue), amphiregulin (serum protein and mRNA), *betacellulin* (mRNA), IGF-1R (protein), PTEN (protein), transforming growth factor alpha (TGFα) (serum protein), epidermal growth factor (EGF) (serum protein), serum HER2/shed HER2 (sHER2), amplifications of *c-Myc* or topoisomerase 2A (*TOP2A*), and mutations in phosphatidylinositol-4,5-bisphosphate 3-kinase catalytic subunit alpha (*PIK3CA*)*.* Here we report the results of the biomarker analysis.

## Materials and methods

### Study design

**Figure 1:**
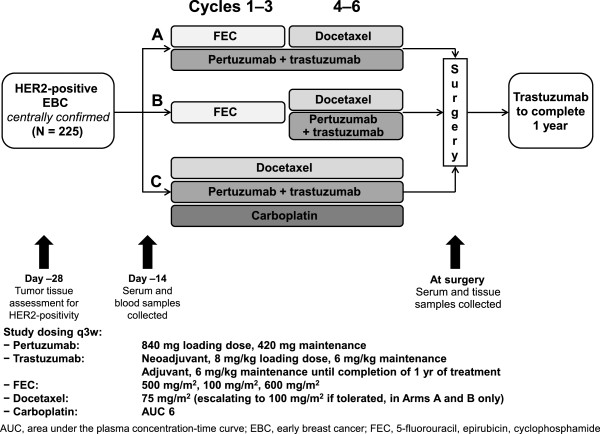
**The TRYPHAENA study design.** (Biomarker assessments are indicated by arrows).

The details of the trial design and patient population have been previously reported [21]. Briefly, 225 patients were recruited to the TRYPHAENA study from 44 centers in 19 countries; 73 patients were randomized 1:1:1 to Arm A (six cycles of pertuzumab plus trastuzumab, with 5-fluorouracil/epirubicin/cyclophosphamide (FEC) for cycles one to three and docetaxel for cycles four to six), 75 to Arm B (FEC for cycles one to three followed by pertuzumab plus trastuzumab with docetaxel for cycles four to six), and 77 to Arm C (six cycles of pertuzumab plus trastuzumab with docetaxel and carboplatin) (Figure 1). The study was conducted in full accordance with the guidelines for Good Clinical Practice and the Declaration of Helsinki. Written informed consent was obtained from each participant. Approval for the protocol and for any modifications was obtained from independent ethics committees (committees are listed in Additional file 1). Baseline demographics were generally well balanced across arms (Table S1 in Additional file 2).

### Biomarker assessments

Tumor samples (either core biopsies or incisional biopsies) were taken from all patients at baseline. Biopsies of the primary tumor were collected for central confirmation of HER2 status and assessment of candidate markers in tumor tissue. Tumor samples from patients with residual disease were also collected at definitive surgery (Figure 1).Serum samples (4.9 mL whole blood per sample) for biomarker assessment were collected at baseline and at the time of definitive surgery (Figure 1).

### Biomarker analysis

#### Immunohistochemistry (IHC)

IHC for HER2, HER3, IGF-1R, and PTEN was performed (by Targos Molecular Pathology GmbH, Germany) on 4 μm tumor tissue sections. HER2 levels were assessed using the Ventana Pathway 4B5™ kit (Ventana Medical Systems, USA) as per the manufacturer’s instructions. All other proteins were detected using commercially available monoclonal antibodies (CONFIRM™ anti-IGF-1R (G11) rabbit monoclonal primary antibody, (Ventana Medical Systems, USA); anti-human EGFR clone 3C6, (Ventana Medical Systems, USA); mouse anti-human HER3 clone DAK-H3-1C, code M7297, (Dako, Denmark); 138G6 PTEN, (Cell Signaling, USA). Protein expression was evaluated semiquantitatively by several pathologists using multicategorical, exploratory IHC scoring. Compulsory assessment for pathologists participating in the study ensured high levels of inter- and intra-reader concordance. Inter-reader concordance at the central laboratory is >99% for HER2 with only 0.76% samples re-scored after reassessment. Local preselection based on HER2 status was carried out and HER2 results were confirmed by the central laboratory prior to patient randomization. For each biomarker assessed by IHC, a modified H-score [23] was derived by classifying the intensity of staining according to three categories (1+, 2+, 3+) and assessing the percentage of tumor cells within each staining intensity (P1, P2, P3, respectively). A modified H-score was calculated as follows: 1 was added to each staining intensity (1+, 2+, and 3+) and then multiplied by the corresponding percentage of tumor cells (P1, P2, and P3). The percentage of cells stained with an intensity of 0 was used only as a quality-control check. The modified H-score was therefore defined as:

$$\begin{array}{l} \mathrm{Modified}\;H‒\mathrm{score}\;=\;\left(1+1\right)\times P1\;+\;\left(2+1\right)\times P2\;+\;\\ \;\left(3+1\right)\times P3. \end{array}$$

This score has a maximum value of 400. In cases where no staining was observed on the entire tissue section a score of 0 was given. A minimum of 10 viable tumor cells were counted. For each sample, staining intensities and patterns per cell compartment (that is, membrane, cytoplasm, nucleus) were assessed separately. The modified H-score (hereafter referred to only as H-score) was calculated for those cell compartments for which specific staining was identified and a biologic rationale for the subcellular location of the respective marker existed (for example, for PTEN, nuclear staining is reported to contribute specifically to its tumor suppressor function).

#### Quantitative real time-polymerase chain reaction (qRT-PCR)

To assess mRNA expression of *HER2*, *HER3*, *EGFR*, *amphiregulin*, *and betacellulin*, 200 ng RNA was isolated from tumor tissue sections. qRT-PCR was performed, on the LightCycler™ 2.0 instrument (Roche Diagnostics, Switzerland), using the LC pertuzumab RT-PCR kit, according to in-house (Targos Molecular Pathology GmbH, Germany) specifications.

#### Mutational assay

The mutational status of *PIK3CA* was assessed at Translational Research Sciences (TRS), Roche, Switzerland, using a TaqMan-based assay (Roche Diagnostics, Switzerland) that detects mutations at four hotspots in exons 7, 9, and 20. The eight mutations detected are: 420R, 542K, 545K, 545A, 545G, 1047R, 1047L, and 1047Y.

#### Fluorescence in situ hybridization (FISH)

FISH analysis of *HER2*, *c-Myc*, and *TOP2A* gene amplification status was carried out using commercially available kits and probes (HER2 FISH pharmDx™ Kit, MYC/CEN-8 FISH Probe Kit and TOP2A FISH pharmDx™ Kit respectively, all from Dako, Denmark).

#### Immunoassays

Immunoassays based on an enzyme-linked immmunosorbent assay (ELISA) principle were performed using the immunological multi-parameter chip technique (IMPACT) (Roche, Switzerland). Serum samples were analyzed in duplicate on the CHIP R-ONC 1.02 chip for determination of amphiregulin, EGF, TGFα, and sHER2 (HER2 ECD). The average concentration of two replicates was reported for each sample. Samples below the level of quantification were excluded from the statistical analyses. (Similar results were obtained if these samples were instead coded as 0).

All assays were carried out using the manufacturer’s instructions where available and were otherwise fully validated ‘in-house’ to ensure high-quality, sensitive readouts and robust reproducibility of the assays. Additional methods information is included in Additional file 3.

### Statistical and analytical methods

The basic statistics and interdependencies of the different markers were investigated descriptively using summary statistics, scatter plots, and box plots. For correlative analyses the patient population was divided in two subgroups per marker, a ‘marker high’ and a ‘marker low’ subgroup. The cut point which defined the two subgroups was set as the median for each marker, with the exception of the following: c-Myc, a *c-Myc*:chromosome 8 signal ratio of ≥2.0 was defined as ‘high’ as this signifies gene amplification; TOP2A, a *TOP2A*:chromosome 17 signal ratio of ≥2.0 was defined as ‘high’; *PIK3CA* mutations, groups were defined based on the presence or absence of any mutation (yes/mut versus no/WT), ‘WT’ being assigned only to samples in which all reactions gave a valid result for ‘No mutation’, whereas if one or more reactions failed, that sample was classified as ‘NA’. The rationale for using the median as the cut point definition (for most markers) is that unlike the mean, the median is largely at the highest density (mode); therefore, in an exploratory setting, the median serves the purpose of identifying high and low subgroups until the ‘true’ cut point is known. The median cut point is also the least sensitive to abnormalities in data, such as when the data are skewed from normal distribution. As the study design does not include a control arm (that is, without pertuzumab administration), it was not possible to identify markers of response to pertuzumab. Therefore, within-arm analyses comparing patients with a pathologic complete response (pCR) (ypT0/is) with patients not achieving a pCR were performed for each biomarker studied. By pooling treatment arms, the overall study population was also assessed. Pooling the data from all three arms ensured sufficient data were available, particularly when considering the more stringent definitions of pCR; however, the analyses presented here are considered exploratory and *P* values should not be used to draw definitive conclusions.

## Results

### Baseline biomarker levels

The proportion of patients with valid biomarker measurements ranged from 48% to 97% for the biomarkers analyzed with the exception of HER2 membrane by IHC, which was collected for 100% of patients (as it was required for study inclusion). The variability in data availability was more apparent for biomarkers measured in tissue (48% to 93% (excluding HER2)) than in serum samples (90% to 97%) due to technical failure, mainly attributed to suboptimal tissue quality or limited availability of tumor tissue.

At baseline, the expression levels of the markers analyzed were well balanced across the three treatment arms with the exception of median HER3 protein and mRNA levels, which were lower in Arm B than in Arms A and C (Table S2 in Additional file 2). Since analyses were generally performed within arms rather than between arms, however, this does not impact overall conclusions.

For both HER2 and PTEN, the majority of patients had an H-score at exactly the median cut point of 400 or 200, respectively, resulting in unequal distribution between the ‘high’ and ‘low’ subgroups as only a few patients were categorized as ‘low’.

Although betacellulin and amphiregulin mRNA levels were above the specified limit of detection, the quantities detected by qRT-PCR were very small in the majority of patients. Therefore, the signal strength for the mRNA for these markers was too weak to allow for meaningful data interpretation particularly in the case of betacellulin. Hence betacellulin was not included in the correlative analyses as observed differences were still within the variance of the assay, while data for amphiregulin that are shown have to be interpreted with caution.

The EGFR protein expression levels as detected by IHC were also generally low, with no expression detected in the majority of cases, making data interpretation difficult.

### Correlation of baseline biomarker levels with clinical outcome (pCR, ypT0/is)

**Figure 2:**
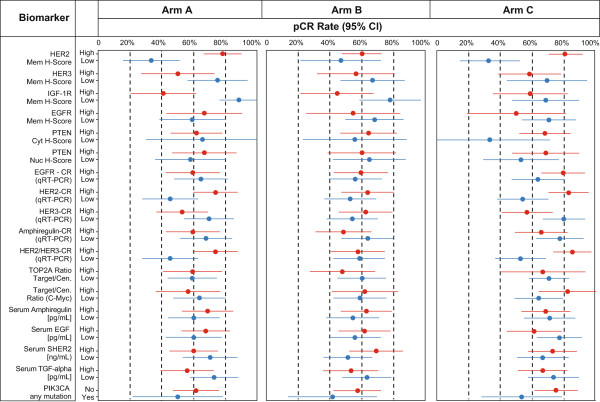
**Analysis by treatment arm of the relationship between biomarkers and pathologic complete response (pCR), adjusted for estrogen receptor status and breast cancer type (operable, locally advanced or inflammatory breast cancer).** Forest plots represent the pCR rate (circle) with 95% confidence limits (line). The high or low categorization indicates whether biomarker levels were above or below the median cut point. cen, centromere; CR, concentration ratio; cyt, cytoplasmic; mem, membrane; nuc, nuclear.

**Figure 3:**
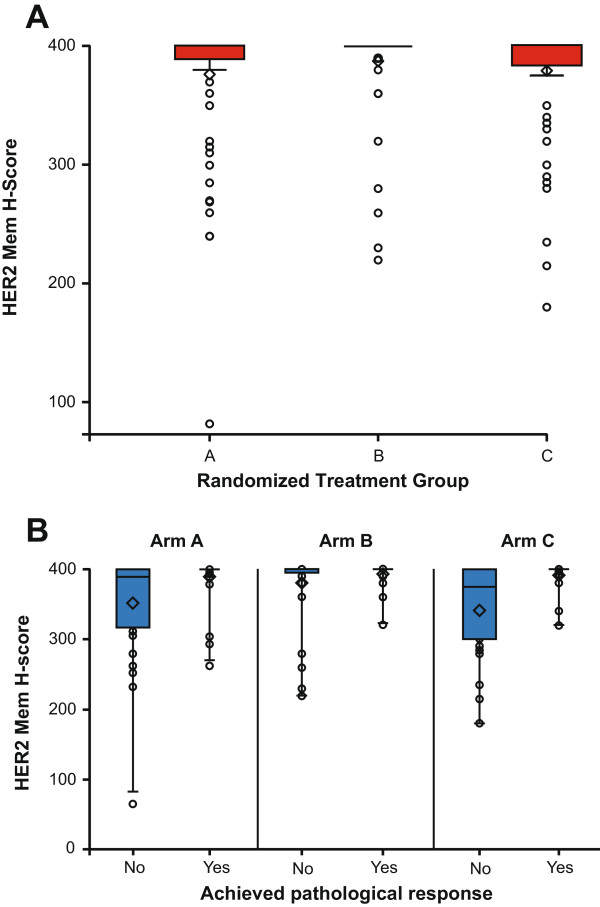
**The relationship between human epidermal growth factor receptor 2 (HER2) expression and efficacy. (A)** Baseline HER2 membrane H-score by treatment arm (Arm A n = 73, Arm B n = 75, and Arm C n = 77); **(B)** Box plot to describe the relationship between baseline HER2 membrane H-score and achievement of pathologic complete response by treatment arm (in the box plot, the horizontal line represents the median value, the diamond represents the mean, the upper and lower bounds of the box represent the 75th and 25th quartiles, respectively, and whiskers represent 95% confidence limits).

pCR (ypT0/is (no evidence of invasive tumor residues in the breast)) rates were compared between patients in the ‘high’ or ‘yes’ subgroups for each biomarker versus the ‘low’ or ‘no’ subgroups, for all treatment arms separately or pooled. For the pooled analyses the chi-squared test was used to explore whether differences observed were statistically significant when adjusted for estrogen receptor (ER) status (Table 1). Adjustment for ER status was important to rule out potential bias in data interpretation introduced by ER status since pCR rates were considerably lower in patients with ER-positive disease versus those in the ER-negative group [21]. Figure 2 shows the unadjusted data per arm.The population enrolled in the TRYPHAENA study was centrally selected for HER2 overexpression and in general showed very high HER2 expression levels. Almost all patients reached the maximum level as measured by the modified H-score with a very narrow dynamic range in general, most prominent in Arm B (Figure 3A). This fact has to be taken into account particularly when interpreting HER2-related data. Of the markers analyzed, HER2 levels (protein and mRNA) showed an association with pCR rates when data from all arms were pooled. With almost no numerical difference in the HER2 protein levels between those above and below the median, comparison of truly different patient populations with regard to HER2 expression is not possible. In Arm B such association was not observed for HER2 protein or for HER2 mRNA levels but the comparison of the two unevenly sized groups (60 (high) versus 15 (low)) makes accurate interpretation difficult. When comparing baseline HER2 expression in patients who later went on to achieve a pCR to expression in those who did not, levels of expression were generally more variable in the group of patients who did not go on to achieve a pCR (Figure 3B). There was, however, a substantial overlap in expression levels between the two groups, which did not allow identification of a meaningful cutoff point to discriminate between the responder and nonresponder groups.

**Table 1:** The relationship between biomarker levels and whether a pathologic complete response (pCR) was achieved, adjusted for estrogen receptor status (all arms pooled)

Biomarker*	**Patients achieving a pCR in the ‘high’ or ‘yes’ subgroup, n/N (%)**	**Patients achieving a pCR in the ‘low’ or ‘no’ subgroup, n/N (%)**	***P *** **value (chi-squared test)**
HER2 membrane	115/159 (72.3)	24/66 (36.4)	0.00002
HER3 membrane	32/58 (55.2)	38/54 (70.4)	0.32864
IGF-1R membrane	27/57 (47.4)	44/56 (78.6)	0.05313
EGFR membrane	21/36 (58.3)	48/73 (65.8)	0.01037
PTEN cytoplasmic	59/92 (64.1)	11/21 (52.4)	0.04968
PTEN nuclear	39/60 (65.0)	31/53 (58.5)	0.22086
*EGFR* mRNA	65/98 (66.3)	60/98 (61.2)	0.65169
*HER2* mRNA	78/106 (73.6)	53/104 (51.0)	0.00060
*HER3* mRNA	60/105 (57.1)	71/105 (67.6)	0.89283
*Amphiregulin* mRNA	55/95 (57.9)	66/95 (69.5)	0.42068
*TOP2A*	35/62 (56.5)	83/130 (63.9)	0.57728
*c-Myc*	40/61 (65.6)	66/106 (62.3)	0.52483
Amphiregulin	68/102 (66.7)	62/101 (61.4)	0.29743
EGF	65/102 (63.7)	65/101 (64.4)	0.83132
sHER2	68/102 (66.7)	62/101 (61.4)	0.89167
TGFα	60/102 (58.8)	70/101 (69.3)	0.16475
PIK3CA mutation	19/39 (48.7)	81/126 (64.3)	0.17160

*All biomarkers assessed by IHC are represented by H-scores. EGF*,* epidermal growth factor; EGFR*,* epidermal growth factor receptor; HER*,* human epidermal growth factor receptor; IGF-1R*,* insulin-like growth factor 1 receptor; PIK3CA*,* phosphoinositide-3-kinase, catalytic alpha polypeptide; PTEN*,* phosphatase and tensin homolog; sHER2*,* serum HER2; TGFα, transforming growth factor alpha; TOP2A*,* topoisomerase 2 alpha.

EGFR membrane and PTEN cytoplasm H-scores showed significant correlations with pCR rates in Arm C only (the anthracycline-free arm). When arms were pooled the correlation was significant, as described by chi-squared test (*P* = 0.0157). (High EGFR membrane expression correlated with lower pCR rates, whereas the opposite was true for PTEN cytoplasm scores). However, these results were limited by the inconsistency observed between arms and by there being too few patients in the ‘low’ subgroup for PTEN (six to nine per arm) to allow any conclusions to be drawn. Complete absence of PTEN, which is biologically the most meaningful cutoff was only observed in five patients therefore not allowing correlative analyses. Similarly, for EGFR, protein expression levels were generally very low or absent in the majority of cases; therefore, the number of patients in the ‘high’ group (10 to 15 per arm) was too low to allow reliable analysis and correlations were not consistent across arms. For IGF-1R membrane H-score when adjusted for estrogen receptor status, there was no significant difference in pCR rate between patients in the high and low subgroups. *TOP2A* amplification status did not appear to be associated with a differential benefit in patients receiving anthracycline treatment. In patients carrying a *PIK3CA* mutation, a numerically lower pCR rate was observed compared to patients whose tumors expressed wild-type *PIK3CA*. This was seen in all treatment arms but did not reach statistical significance. In a per-exon analysis, no difference was seen between mutations in exon 9 and mutations in exon 20 regarding their association with pCR rates. No exon 7 mutations were detected in the TRYPHAENA population (Table 2).

**Table 2:** ***PIK3CA *** **mutation-dependent responsiveness to pertuzumab treatment (all arms pooled)**

***PIK3CA *** **exons**	**Number of patients achieving a pathological complete response (pCR) per-exon mutation/total number of patients with a mutation in that exon (population pCR rate 139/225 (62%))**
Exon 7	0/0
Exon 9	5/11
Exon 20	14/28

### Correlation of baseline biomarker levels with pCR by different definitions (ypT0/is, ypT0, ypT0/is ypN0, ypT0 ypN0)

For the majority of biomarkers, any correlation or lack of correlation with pCR remained consistent regardless of the pCR definition applied (ypT0/is, no evidence of invasive tumor but noninvasive tumor may be present (that is, carcinoma *in situ*); ypT0, no evidence of invasive tumor; ypT0/is ypN0, no evidence of invasive tumor, no regional lymph node metastasis (noninvasive tumor may be present); ypT0 ypN0, no invasive or noninvasive tumor, no regional lymph node metastasis). (Data not shown.) The only exceptions observed were: PTEN H-score in cytoplasm was not associated with pCR except by the ypT0 ypN0 definition in Arm C; no significant association between pCR and HER2 mRNA levels was seen in Arm A for the ypT0 and ypT0 ypN0 definitions; HER2:HER3 levels were significant in Arm C for ypT0 and ypT0 ypN0 definitions; the incidence of *PIK3CA* mutations showed no correlation with pCR overall for the ypT0/is ypN0 definition unlike the other definitions and if the data were not pooled then a significant association was observed only in Arm B with the ypT0 ypN0 definition.

### The relationship between pCR and biomarker changes following treatment

**Figure 4:**
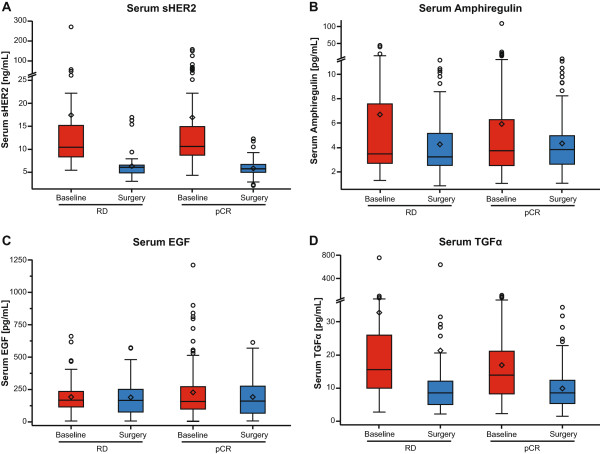
**Analysis of serum biomarker levels at baseline and at surgery, by pathologic complete response status. (A)** sHER2, **(B)** amphiregullin, **(C)** EGF, **(D)** TGFα. (n = 112 for pCR (responders) and n = 56 for residual disease (RD) (nonresponders)) (in the box plot, the horizontal line represents the median value, the diamond represents the mean, the upper and lower bounds of the box represent the 75th and 25th quartiles, respectively, and whiskers represent 95% confidence limits). EGF, epidermal growth factor; pCR, pathological complete response; (s)HER2, (serum/shed) human epidermal growth factor receptor 2; TGFα, transforming growth factor alpha.

Matched pair analyses were carried out to evaluate the effect of treatment on biomarker levels in both serum and tissue samples. In serum samples, sHER2 and TGFα levels substantially decreased during treatment, whereas both amphiregulin and EGF levels remained similar irrespective of treatment (Figure 4). When changes in biomarker levels occurred between baseline and time of surgery, these were similar regardless of whether patients had a pCR or not. Any changes observed were consistent across all treatment arms.

**Figure 5:**
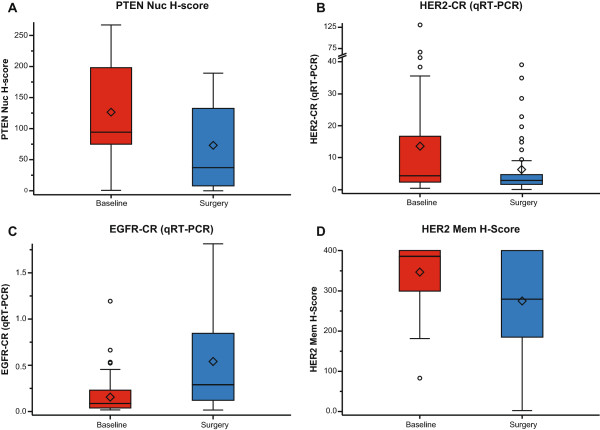
**Comparison of baseline levels of biomarkers derived from tissue samples with the levels detected at surgery. (A)** PTEN nuc, **(B)** HER2-CR, **(C)** EGFR-CR, **(D)** HER2-mem. (In the box plot, the horizontal line represents the median value, the diamond represents the mean, the upper and lower bounds of the box represent the 75th and 25th quartiles, respectively, and whiskers represent 95% confidence limits). CR, concentration ratio; EGFR, epidermal growth factor receptor; HER2, human epidermal growth factor receptor 2; mem, membrane; nuc, nuclear; PTEN, phosphatase and tensin homolog.

Analyses of tissue samples revealed that PTEN nuclear H-score and *HER2* mRNA levels substantially decreased between baseline and surgery. *EGFR* mRNA levels were observed to increase significantly during the same period, although overall levels were very low (Figure 5). The median HER2 membrane H-score was somewhat reduced at time of surgery, but this observation was not statistically significant and in the majority of cases did not impact the overall IHC scoring. In nine patients, this led to a change in HER2 status (that is, HER2-positive to HER2-negative disease) although this does not necessarily equate to a complete loss of HER2 expression. Other markers remained unchanged. As these tissue analyses are only possible in patients not achieving pCR, no judgment can be made regarding whether those changes are characteristic for sensitivity to treatment.

### Correlation of biomarker levels with tumor size

In addition to analyzing association of biomarkers with pCR as an endpoint, the correlation of biomarkers with tumor size (degree of tumor shrinkage) was also measured as an alternative endpoint. No correlations were found between the level or presence of any biomarker and tumor shrinkage as a result of treatment (data not shown).

## Discussion

In the TRYPHAENA study, a range of biomarkers and their association with pCR were assessed. Although the biomarker analysis methods used at the time of this investigation were considered ‘state of the art’, newer methods such as quantitative IHC or immunofluorescence techniques have since come into common use. The use of these techniques (where appropriate) may be of value to similar studies in the future given the greater degree of quantification possible.

According to our analyses, no subpopulation within the patients with HER2-positive disease in TRYPHAENA could be identified who might derive the greatest benefit from pertuzumab-containing regimens or who should not be treated with pertuzumab, since patients in all arms received pertuzumab therapy. In TRYPHAENA, HER2 expression at both the protein and mRNA levels were the only biomarkers associated with pCR rate. This correlation was not observed in Arm B, which may be attributed to the fact that in Arm B the treatment duration was shorter, with only three cycles of HER2-targeted therapy compared with six cycles in the other two treatment arms. Applying the current scoring criteria, HER2 expression remains the only marker suitable for patient selection at present.

It should be taken into account that the patients enrolled in TRYPHAENA showed relatively high HER2 protein expression levels, with a very narrow dynamic range and little numerical difference in the protein levels between those above and below the median. As the majority of tumors expressed HER2 at the maximum detectable level of H-score 400, two unequally sized groups (70% versus 30% of the patients) were compared as the median of 400 was used as cut point. Therefore, the HER2 expression levels at the cut point that were identified in this population to be associated with pCR cannot likely define a clinically meaningful and biologically distinct subpopulation.

When data were pooled for all three arms, statistically significant correlations of biomarker status with pCR also occurred with EGFR (membrane H-score) and PTEN (cytoplasm H-score); however, there were only a small number of patients in the PTEN ‘low’ subgroup (owing to the majority of patients falling exactly on the median cut point) and in the EGFR ‘high’ subgroup (owing to low expression levels across the population), which limits the interpretation of these results. In addition, significant association of EGFR and PTEN expression with pCR status was observed in Arm C but not Arms A and B, although there is no biologic rationale as to why an association would be attributable to a different chemotherapy backbone or sequence thereof rather than HER2-targeted therapy. These associations may therefore be somewhat artificial or biased due to the small numbers of patients and multiple analyses performed. Future trials with larger samples sizes are needed to explore the robustness of the signal.

Previous analyses have noted that *TOP2A* amplification in HER2-positive patients was a predictor of extended PFS and disease-free survival (DFS) for patients who received anthracycline therapy with or without trastuzumab [24]. In TRYPHAENA, no association was identified between expression levels of *TOP2A* and pCR rate in either the anthracycline-containing (Arms A and B) or anthracycline-free (Arm C) arms. This might be due to the additional administration of pertuzumab in TRYPHAENA, unlike in the study by Press *et al*. [24]. As the addition of pertuzumab significantly increases pCR rate [10] any additional anthracycline-specific benefit might be masked, or perhaps a lack of efficacy of anthracyclines in some patients might be overcome by treatment with two anti-HER2-targeted antibodies. Alternatively, the difference may be due to the different outcome measures used or to our limited sample size. Press et al. [24] considered the association of *TOP2A* amplification and improved PFS or DFS with anthracycline-containing therapy, that is, looking at long-term benefit, whereas TRYPHAENA explored the relationship between *TOP2A* amplification and pCR rate with anthracycline-containing treatment and analyzes tumor response as a shorter-term response measure.

Mutations in exon 9 of *PIK3CA* have been linked to a lack of sensitivity, that is, reduced pCR rate in patients receiving HER2-targeted therapy with trastuzumab, pertuzumab, or both [10]. In TRYPHAENA, generally, numerically lower pCR rates were observed in patients with tumors carrying any *PIK3CA* mutation tested for, although this did not reach a statistically significant level. As this was consistent across arms and in the absence of a control arm, it was not possible to correlate this to HER2-targeted treatments. No correlation between specific *PIK3CA* mutations and pCR was observed in TRYPHAENA, though the sample sizes in TRYPHAENA would have limited the power to detect specific correlations.

A number of different pCR definitions were applied during data analysis and, in general, it was found that the results were consistent regardless of the definition used. The small differences in pCR rates according to different definitions highlight the need for consistency of pCR definition use across studies to allow meaningful comparison. pCR defined by ypT0 ypN0 has recently been associated with the most favorable outcomes following neoadjuvant chemotherapy compared with less stringent pCR definitions [25].

Although changes in serum biomarker levels were observed between baseline and surgery, these were not associated with pCR and therefore could not be defined as markers of response. Some changes in biomarker levels in tumor tissue from baseline to surgery were observed, but these cannot be correlated to outcome owing to the lack of tissue availability from patients achieving pCR. The observed reduction in HER2 mRNA may indicate early changes that are not yet reflected in HER2 protein levels. This could be due to the short treatment duration or may indicate that the protein remains stable despite changes in mRNA levels. No conclusion can be drawn on whether such changes predict for nonresponse to pertuzumab-containing regimens.

In addition, we considered tumor size as an alternative clinically meaningful endpoint to pCR for correlative analyses with biomarkers. However, no biomarker tested was found to correlate with tumor shrinkage.

## Conclusions

The results from the TRYPHAENA study, consistent with findings from the NeoSphere [10] and CLEOPATRA [11] trials, emphasize the importance of HER2 as the most clinically relevant biomarker for selecting patients for HER2-targeted therapy in the absence of signals for other markers analyzed. The CLEOPATRA study also found that although mutations in PIK3CA were not associated with resistance to pertuzumab, PIK3CA mutational status may identify patients with poorer prognoses and particular unmet medical needs suggesting that clinical trials of HER2-targeted molecules in combination with PI3K pathway-targeted agents may therefore be justified [11]. Additionally in the NeoSphere Study, pCR following anti-HER2 antibody-based therapy was associated with high expression of one or more of interferon gamma (IFNγ), signal transducer and activator of transcription 1 (STAT1), major histocompatibility complex 2 (MHC2), cluster of differentiation 8A (CD8A) and/or programmed cell death-1 (PD1) [26], suggesting that adaptive immune mechanisms might modulate the benefit from HER2-directed therapy. Additional biomarker analyses from ongoing studies such as NeoALTTO [27] may help to detect further biomarkers to predict outcomes for patients receiving HER2-targeted therapy in the neoadjuvant setting.

Currently, HER2 expression remains the only biomarker for predicting outcomes for patients with HER2-positive breast cancer and for basing treatment decisions on in the clinic.

## Abbreviations

(s)HER2: (serum/shed) human epidermal growth factor receptor 2; ADCC: antibody-dependent cellular cytotoxicity; CD8A: cluster of differentiation 8A; DFS: disease-free survival; ECD: extracellular domain; EGF(R): epidermal growth factor (receptor); ELISA: enzyme-linked immunosorbent assay; ER: estrogen receptor; FEC: 5-fluorouracil/epirubicin/cyclophosphamide; FISH: fluorescence *in situ* hybridization; HER3: human epidermal growth factor receptor 3; IFNγ: interferon gamma; IGF-1: insulin-like growth factor 1; IHC: immunohistochemistry; IMPACT: immunological multi-parameter chip technique; MBC: metastatic breast cancer; MHC2: major histocompatibility complex 2; mRNA: messenger RNA; OS: overall survival; pCR: pathological complete response; PD-1: programmed cell death-1; PFS: progression-free survival; PIK3CA: phosphatidylinositol-4,5-bisphosphate 3-kinase catalytic subunit alpha; PTEN: phosphatase and tensin homolog; qRT-PCR: quantitative reverse transcription-polymerase chain reaction; STAT 1: signal transducer and activator of transcription 1; TGFα: transforming growth factor alpha; TOP2A: topoisomerase 2A.

## Competing interests

AS has had consultant or advisory relationships with F. Hoffmann-La Roche Ltd, Medac and Celgene and has received honoraria from F. Hoffmann-La Roche Ltd, Celgene, Eisai, AstraZeneca, GlaxoSmithKline and Novartis. AS has received research funding from F. Hoffmann-La Roche Ltd and Celgene. SC has had a consultant or advisory relationship with, and has received honoraria from, F. Hoffmann-La Roche Ltd. RH has no conflicts of interest to disclose. CT has received honoraria from F. Hoffmann-La Roche Ltd. JR, VM and GR are employees of Roche Products Ltd. VM and GR hold stocks in Roche Products Ltd and GR holds stocks in GlaxoSmithKline. GR is named as inventor on some use patents for pertuzumab for which he receives no remuneration. AK is an employee of Genentech and holds stocks in F. Hoffmann-La Roche Ltd. JC has had a consultant or advisory relationship with, and has received honoraria from, F. Hoffmann-La Roche Ltd, Celgene, Novartis and has received honoraria from Eisai.

## Authors’ contributions

GR, AK and VM contributed to the conception, design, conduct and analysis of the study. JR participated in the conception and design, assembly of data, data analysis and interpretation. AS participated in the design of the study. Acquisition of biomarker samples from patients in the study was carried out by AS, SC, RH, CT and JC. RD reviewed and interpreted biomarker results. All authors critically reviewed drafts of the manuscript and read and approved the final manuscript.

## Supplementary Material

Additional file 1 Ethics committee approval. Click here for file

Additional file 2 Supplementary data: baseline characteristics and biomarker levels. Click here for file

Additional file 3 Supplementary methods. Click here for file
