# Supplementary material for: Evaluating the predictive value of biomarkers for efficacy outcomes in response to pertuzumab- and trastuzumab-based therapy: an exploratory analysis of the TRYPHAENA study
Source: Breast Cancer Res. 2014 Jul 8;16(4):R73. doi: 10.1186/bcr3690 (PMC4226982; doi:10.1186/bcr3690)
Supplement: Additional file 1 — Ethics committee approval. [file bcr3690-S1.docx]

**Additional file 1**

The independent ethics committees that approved the TRYPHAENA protocols and amendments where appropriate were:

CE università Cattolica del S. Cuore -Policl. Gem, Italy;

Comitato Etico A.O. San Gerardo di Monza, Italy;

EK Heidelberg, Germany;

Comisia Nationala de Etica, Romania;

Ethic Committee Institute for Oncology and Radiology of Serbia;

Agency for medicinal products and medical devices, Bosnia and Herzegovina;

Ottawa Hospital Research Ethics Boards; Canada;

UBC BCCA Research Ethics Board (BCCA REB), Canada;

Comitê de Ética em Pesquisa da PUCRS, Brazil;

Comite de Etica em Pesquisa do Centro de Referencia da Saude da Mulher, Brazil;

Northern X Ethics Committee, New Zealand;

Pharma-Ethics Independent Research Ethics committee, South Africa;

Kyungpook National Uni Hospital, Republic of Korea;

TVGH Institutional Review Board, Taiwan;

Comité Etico Hospital Vall de Hebron, Spain;

Comité de Etico Hospital Universitario Puerta de Hierro, Spain;

Western Institutional Review Board, United States;

Hospital Angeles Metropolitano, Mexico;

Southampton & South West Hampshire LREC (B), UK;

Kantonale Ethikkommission Aarau, Switzerland;

Korea University Guro Hospital, Republic of Korea;

Kantonale Ethikkommission Zürich (KEK), Switzerland;

CEIC - Comissão de Ética para Investigação Clínica, Portugal;

Regionala Etikprövningsnämnden i Stockholm, Sweden;

McGill University Health Centre – Research Ethics Board, Canada;

University of Pretoria Research Ethics committee, Faculty of Health Sciences, South Africa;

Comité Etico de Euskadi, Spain;

Comité Ético de Investigación Clinica Hospital Universitario Reina Sofía, Spain;

Central Ethics Committee Agency for medicines and medical devices, Croatia;

IRB,China Medical University Hospital, Taiwan;

Centro Estatal De Cancerologia Dr Miguel Dorantes Mesa, Mexico;

Ministry of Health and Social Welfare, Croatia.
