# Supplementary material for: Evaluating the predictive value of biomarkers for efficacy outcomes in response to pertuzumab- and trastuzumab-based therapy: an exploratory analysis of the TRYPHAENA study
Source: Breast Cancer Res. 2014 Jul 8;16(4):R73. doi: 10.1186/bcr3690 (PMC4226982; doi:10.1186/bcr3690)
Supplement: Additional file 2 — Supplementary data: baseline characteristics and biomarker levels. [file bcr3690-S2.docx]

**Table S1.** Baseline characteristics in the ITT population

|  | FEC+H+P x3 → T+H+P x3 n = 73 | FEC x3 → T+H+P x3 n = 75 | TCbH+P x6  n = 77 |
| --- | --- | --- | --- |
| Median age, years (range) | 49.0 (27–77) | 49.0 (24–75) | 50.0 (30–81) |
| Median weight, kg (range) | 63.3 (44–111) | 64.9 (42–112) | 66.5 (45–128) |
| Race, n (%)  Black  White  Oriental  Other | 4 (5.5) 56 (76.7) 12 (16.4) 1 (1.4) | 3 (4.0) 52 (69.3) 18 (24.0) 2 (2.7) | 2 (2.6) 64 (83.1) 11 (14.3) 0 (0.0) |
| Histologic grade  Well differentiated  Moderately differentiated  Poorly differentiated  Unknown | 3 (4.1) 28 (38.4) 25 (34.2) 17 (23.3) | 2 (2.7) 34 (45.3) 26 (34.7) 13 (17.3) | 2 (2.6) 32 (41.6) 27 (35.1) 16 (20.8) |
| ER-positive and/or PgR-positive, n (%) ER-negative and PgR-negative, n (%) | 39 (53.4)  34 (46.6) | 35 (46.7)  40 (53.3) | 40 (51.9)  37 (48.1) |
| Disease type, n (%)  Operable  Locally advanced  Inflammatory | 53 (72.6) 15 (20.5) 5 (6.8) | 54 (72.0) 17 (22.7) 4 (5.3) | 49 (63.6) 24 (31.2) 4 (5.2) |
| HER2 status by IHC, n (%)  0 and 1+  2+  3+ | 1 (1.4) 5 (6.8)^a^ 67 (91.8) | 0 (0.0) 1 (1.3)^a^ 74 (98.7) | 0 (0.0) 2 (2.6)^a^ 75 (97.4) |
| Primary tumor size at baseline by clinical breast examination, mm  Median (range) | 53 (10–220) | 49 (19–120) | 50 (15–200) |

^a^ All patients with HER2 IHC 2+ status had FISH-positive status

ER, estrogen receptor; FISH, fluorescence in situ hybridization; HER2, human epidermal growth factor receptor 2; IHC, immunohistochemistry; ITT, intent-to-treat; PgR, progesterone receptor

Cb, carboplatin; FEC, 5-fluorouracil, epirubicin, cyclophosphamide; H, trastuzumab; P, pertuzumab; T, docetaxel

**Table S2.** Baseline biomarker levels

| Biomarker | Arm A  n = 73 | | | Arm B  n = 75 | | | Arm C  n = 77 | | |
| --- | --- | --- | --- | --- | --- | --- | --- | --- | --- |
|  | n | median | SD | n | median | SD | n | median | SD |
| HER2  Mem H-score | 73 | 400.0 | 52.8 | 75 | 400.0 | 36.2 | 77 | 400.0 | 45.5 |
| HER3 Mem H-score | 38 | 180.0 | 130.3 | 37 | 50.0 | 136.2 | 37 | 230.0 | 136.5 |
| IGFR-1  Mem H-score | 41 | 180.0 | 144.4 | 36 | 160.0 | 138.8 | 36 | 160.0 | 144.3 |
| EGFR  Mem H-score | 37 | 0.0 | 111.9 | 36 | 0.0 | 103.1 | 36 | 0.0 | 116.4 |
| PTEN Cyt H-score | 40 | 200.0 | 58.0 | 37 | 200.0 | 67.0 | 36 | 205.0 | 66.8 |
| PTEN Nuc H-score | 40 | 150.0 | 95.5 | 37 | 150.0 | 99.5 | 36 | 140.0 | 91.5 |
| EGFR mRNA | 63 | 0.1 | 0.15 | 66 | 0.1 | 2.5 | 67 | 0.1 | 0.4 |
| HER2 mRNA | 69 | 12.6 | 20.6 | 69 | 10.9 | 11.2 | 72 | 10.8 | 14.5 |
| HER3 mRNA | 69 | 0.5 | 0.4 | 69 | 0.4 | 0.5 | 72 | 0.5 | 0.5 |
| Amphiregulin mRNA | 63 | 0.1 | 0.9 | 64 | 0.1 | 1.2 | 63 | 0.1 | 0.5 |
| HER2/HER3 mRNA | 69 | 28.6 | 37.8 | 69 | 23.1 | 40.3 | 72 | 24.9 | 44.6 |
| TOP2A ratio | 66 | 1.6 | 2.5 | 63 | 1.5 | 2.7 | 63 | 1.3 | 2.1 |
| c-Myc ratio | 56 | 1.7 | 3.5 | 55 | 1.5 | 2.2 | 56 | 1.5 | 2.4 |
| Serum amphiregulin | 67 | 3.3 | 3.8 | 70 | 3.5 | 6.8 | 66 | 3.5 | 14.6 |
| Serum EGF | 67 | 158.9 | 198.2 | 70 | 149.0 | 147.8 | 66 | 146.0 | 199.4 |
| Serum HER2 | 67 | 10.8 | 10.6 | 70 | 10.1 | 16.3 | 66 | 10.5 | 38.3 |
| Serum TGFα | 67 | 14.2 | 91.8 | 70 | 12.8 | 10.1 | 66 | 16.5 | 18.5 |
