# Supplementary material for: Evaluating the predictive value of biomarkers for efficacy outcomes in response to pertuzumab- and trastuzumab-based therapy: an exploratory analysis of the TRYPHAENA study
Source: Breast Cancer Res. 2014 Jul 8;16(4):R73. doi: 10.1186/bcr3690 (PMC4226982; doi:10.1186/bcr3690)
Supplement: Additional file 3 — Supplementary methods. [file bcr3690-S3.docx]

**Supplementary methods**

*Sample preparation*

Tissue processing, IHC, FISH, RNA extraction, qRT-PCR and DNA isolation were performed centrally by Targos Molecular Pathology GmbH, Germany. Targos were supplied with, and adhered to, protocols and processing instructions that were developed in-house at Roche Diagnostics. Commercially available assays or kits were employed, where specified, and performed according to the manufacturer’s instructions.

*Validation of biomarker assays*

A number of the assays and antibodies used to assess biomarkers in the TRYPHAENA study were commercially available and have been validated by the manufacturer. Immunohistochemistry was performed on 4 μm tumor tissue sections according to specified instructions that were developed internally and that were aligned with manufacturer’s instructions for the commercially available assays. HER2 levels were assessed using the Ventana Pathway 4B5™ kit. All other proteins were detected using commercially available monoclonal antibodies (IGF-1R, clone G11 from Ventana; mouse anti-human HER3, clone DAK-H3-IC code M7297 from DAKO; anti-human EGFR clone 3C6 from Ventana; PTEN, clone 138G6 from Cell Signaling).
In some cases, additional ‘in-house’ validation of assays was required. For the HER3 antibody, paraffin-embedded cell pellets obtained from cell lines transfected to express either HER1, HER2, HER3 or HER4 were used to show specific staining of HER3 transfected cells with no or minimal staining for HER1 and HER2 and no staining for HER4. Corresponding Western blot experiments showed specific reactivity of the antibody with the HER3 protein and no cross reactivity with other HER family proteins.
Specificity of the IGF-1R antibody was confirmed by peptide inhibition studies where incubation with a synthetic IGF-1R peptide was observed to inhibit antibody binding to IGF-1R in tissue (also see package insert). Further specificity and sensitivity analyses were performed based on normal and neoplastic tissue as described in the package insert.
The specificity of the PI3K assay (TaqMan based technology) was tested using WT cell lines and a plasmid containing the PIK3CA pseudogene sequence. Cross-reactivity between mutations was tested with mutation-specific plasmids. The sensitivity/limit-of-detection of the assay was determined using mutant plasmid blended with WT cell line DNA to assess the effect of DNA input and mutation percentage. Sensitivity and specificity were further evaluated in a method correlation between the PIK3CA assay and Sanger sequencing of 144 breast cancer samples. A subset of the specimens was confirmed by 454 sequencing.
For the qRT-PCR assays for HER1, HER2, HER3, amphiregulin and betacellulin which were performed on a Roche LightCycler 2.0, the assay linear range (upper and lower limit of quantitation) was determined using a dilution series of RNA. This RNA was extracted from formalin-fixed paraffin-embedded tissue (FFPET) specimens or was generated in-vitro. Both intra- and inter-assay reproducibility were assessed using RNA extracted from multiple FFPET sections derived from three cancer patients. Specificity of the assay for RNA over DNA was demonstrated using human genomic DNA.
